# Preferred Source and Perceived Need of More Information about Dental Implants by the Undergraduate Dental Students of Nepal: All Nepal Survey

**DOI:** 10.1155/2018/6794682

**Published:** 2018-03-11

**Authors:** Arati Sharma, Bidhan Shrestha, Bijay Kumar Chaudhari, Pramita Suwal, Raj Kumar Singh, Surya Raj Niraula, Prakash Kumar Parajuli

**Affiliations:** ^1^Department of Prosthodontics, CODS, B.P. Koirala Institute of Health Sciences, Dharan, Nepal; ^2^Department of Prosthodontics, Kantipur Dental College and Hospital, Kathmandu, Nepal; ^3^Department of ENT, NAMS, Bir Hospital, Kathmandu, Nepal; ^4^Department of Public Health and Community Medicine, B.P. Koirala Institute of Health Sciences, Dharan, Nepal

## Abstract

**Objectives:**

This study was conducted to know the preferred source and perceived need of more information about dental implants by the undergraduate students of Nepal and their association with academic levels and gender.

**Materials and Methods:**

It was conducted in all the dental colleges of Nepal from June 2016 to June 2017 after taking ethical clearance and approval from the research committee of BPKIHS. It included all those who were present at the time of survey. Data collection was done through a cross-sectional questionnaire survey during the academic schedule of the colleges, supervised and monitored by the investigators themselves. The collected data were coded and entered in Microsoft excel 2013, and statistical analysis was done by SPSS 20 version.

**Result:**

A majority of the respondents agreed that they were not provided with sufficient information about implant treatment procedures during their BDS program (65.3%), would like more to be provided in the curriculum (95.1%), and would like to get additional reliable information from dental consultants and specialists (40.7%) and training on it from fellowship programs conducted by universities (39.2%). Significant association was seen between the responses and academic levels.

**Conclusion:**

Undergraduate dental students of Nepal want more information about dental implants through various means.

## 1. Introduction

Loss of dentition in the elderly and middle-aged is still prevalent in modern society [1]. The goal of modern dentistry is to maintain normal function, speech, aesthetic, and health of the patient [2]. These aims are achieved by oral implants, which make them a unique treatment option. The literature supports that dental implants have a high success rate and good predictability when used in partially or completely edentulous patients for rehabilitation [2].

Implant therapy is an elective dental procedure [3]. Patient should be informed regarding this therapeutic option, the cost-benefit ratio, complications, and other treatment options available for that particular patient's dental needs. Then, the patient can make an informed decision as to which treatment modality meets his/her clinical needs and financial ability.

In Austrian population, a survey showed that the primary source of patient information on dental implants was dentists followed by friends and acquaintances, media, and the general practitioners. Most of these prospective patients were interested in getting information from dentists [4]. In a survey conducted in India, the result showed that dentists are considered as the main source of information and people want to get information about dental implants from dentists [5]. There are numerous articles published in indexed journals regarding awareness and source of information of patients about dental implants [5–10], whereas very few articles were found on attitude of dental students toward dental implants [11–14].

BDS is a dental degree and stands for Bachelor of Dental Surgery. It is a five and a half years course including one year of compulsory internship. There is strong evidence in the literature that the main source of information about dental treatment modalities to the dental patients is dentists. Increased awareness among the general population indicates that there is a need to provide sufficient basic knowledge about dental implants to the undergraduate students which will help them to deal with those patients during their clinical postings. So, it becomes important to gauge the viewpoint of undergraduate students about the need of more information and preferred sources of information so that programs can be implemented as per interest.

## 2. Materials and Methods

This study was conducted in all the dental colleges of Nepal from June 2016 to June 2017 after taking ethical clearance and approval from the research committee of BPKIHS. The study population was all the undergraduate dental students of Nepal from 1st year to 5th year (excluding interns), and the sample included all those students who were present at the time of survey and excluded those who were absent (consecutive sampling).

Data were collected through a cross-sectional questionnaire survey. Preused questionnaire was taken [14]. A pilot study was carried out. Minor modification was made in the questionnaire. Data collection was carried out during the academic schedules of the colleges, and an effort was made to include maximum number of students. It was supervised and monitored by the investigators themselves. The collected data were coded and entered in Microsoft excel 2013, and statistical analysis was done by SPSS 20 version. Descriptive analysis was done, and chi-square test was applied to see the association of the responses with academic levels and gender.

## 3. Results and Discussion

The total number of students was roughly calculated to be 2400. A total of 1850 questionnaires were distributed, of which 1700 responses were received. Thus, the response rate was 70.83% (dropout rate = 29.16%). All the questionnaires were completely filled. The respondents of our survey were overwhelmingly females (Table 1). A similar trend with the higher number of female respondents was also seen in an all India survey [14]. There is a wave of women dentists surging through dentistry, and the expansion of the number of women in dentistry has been one of the major trends seen in the past few decades.

A total of 4 questions were asked on their preference of source and perception of need of more information about dental implants at each level of their BDS course from 1st year to 5th year.

A majority of the total respondents agreed that they were not provided with sufficient information about implant treatment procedures during their BDS program and would like more information to be incorporated in the curriculum. This was similar to the result of an all India survey, which included interns only [14]. There was no significant association of the responses with gender, but there was a significant association with academic levels (Tables 2 and 3).

Among those who said “Yes” (i.e., they were provided with sufficient information about implant treatment procedures during their BDS program), the highest percentage was of 5th year, and among those who said “No,” the highest percentage was of 3rd year.

Among the respondents who said “Yes” (i.e., they would like more information about implant treatment procedures to be provided in the BDS curriculum), higher percentages of students were of 4th year and 5th year; among those who said “No,” the highest percentage was of 3rd year. The reason behind this may be that, for the students below 4th year, the information given about dental implants may have been perceived as sufficient for that level.

When asked from where they would like to get additional reliable information about dental implants, there were differences in the mind-set of the students with lack of any definitive consensus, and those differences in the mind-set were more among the students in the 5th year. Highest number of students said that they would like to get it from dental consultants and specialists (Table 4), whereas, in an all India survey, a majority of the interns said that they would like more reliable information from one-year certificate or module-based courses conducted by colleges or trained implantologists [14]. This difference in the result of the two studies may be due to the difference in the academic levels of the students.

There was a significant association of the response with the academic level. Upto 4th year, highest percentages of the students said “dental consultants and specialists,” whereas those from the 5th year said “one-year certificate or module-based courses conducted by colleges or trained implantologists” (Table 4).

In one study on the outlook of undergraduate dental students on dental implants, 33.12% of the students who were aware of dental implants said that their source of information were their dentists, and out of those students, 10.1% were of 1st year, 10.7% were of 2nd year, 38.4% were of 3rd year, and 40.9% were of 5th year. In the same study, 50% of the students learnt about dental implants from books, magazines, and electronic media [13].

When asked from where they would like to receive training on dental implants, again there were major differences in their mind-set and no definitive consensus was reached. The differences were more within the 5th year students (Table 5). The highest percentage of the total respondents who agreed on a single option was only 39.2% who said “fellowship programs conducted by universities.” Again, there was a significant association of the response with the academic level. Upto 4th year, highest percentages of the students said, “fellowship programs conducted by universities,” whereas, highest percentage of the 5th year students said, “one-year certificate or module-based courses conducted by colleges or trained implantologists.”

In the all India study, there was no such major differences in the mind-set of the students and a majority of the students (52.0%) said that they would like to receive training from one-year certificate or module-based courses conducted by colleges or trained implantologists [14]. This difference in the results of the two studies may be due to difference in academic levels.

In one study, over 70% of the subjects felt the need to have implant training as a part of their undergraduate clinical curriculum, whereas 56.6% in the same study felt that it should be made into a separate speciality [11].

In a study on the knowledge, attitude, and practice of the dental and medical practitioners regarding dental implants, 88.1% of the dental practitioners said “Yes” (i.e., they would like to gain more information regarding dental implants and its placement) [15]. Another study concluded that, among the general dental practitioners, their attitudes are not in line with evidence-based knowledge, and variations in their attitudes existed due to variations in the factors like training and experience, years in practice, and place of graduation [16].

## 4. Conclusion

A majority of the students agreed that they were not provided with sufficient information about dental implant treatment procedures and perceived the need of more information to be included in their curriculum. Their preferences of source of information varied, and no definitive consensus was reached on a particular option. The responses were significantly associated with academic levels. Highest percentage of those below 5th year preferred dental consultants and specialists as reliable source of more information, whereas highest percentage of 5th year preferred one-year certificate or module-based courses conducted by colleges or trained implantologists as reliable source. About receiving training on dental implants, highest percentage of those below 5th year preferred fellowship programs conducted by universities, whereas highest percentage of 5th year preferred one-year certificate or module-based courses conducted by colleges or trained implantologists. There was no significant association with gender.

## 5. Limitations

One limitation of this study was that data collection from all the dental colleges of Nepal was done at different periods of their academic year. In some colleges, it was done during the middle of their session, whereas in other colleges, it was done before their annual exams. Another limitation was that unequal number of participants were included at different academic levels.

## Figures and Tables

**Table 1:** Demographic structure of the surveyed population.

Characteristics	Frequency (%)
*Total*	1700 (100.0%)
*Age*	—
Minimum (17 years)	—
Maximum (29 years)	—
Mean ± SD	21.47 ± 2.07
*Sex*	
Male	479 (28.2%)
Female	1221 (71.8%)
*Academic level*	
First year	376 (22.1%)
Second year	316 (18.6%)
Third year	330 (19.4%)
Fourth year	337 (19.8%)
Fifth year	341 (20.1%)

**Table 2:** Overall responses (*N*) to the 1st question (A1) on perceived need of more information and its association with gender and academic levels.

Question		*N* (%)	Gender			Academic year					
			Male, *n* (%)	Female, *n* (%)	*P* value	1st, *n* (%)	2nd, *n* (%)	3rd, *n* (%)	4th, *n* (%)	5th, *n* (%)	*P* value
A1^∗∗^	1	590 (34.7)	167 (34.9)	423 (34.6)	0.487	112 (29.8)	111 (35.1)	92 (27.9)	115 (34.1)	160 (46.9)	<0.001
	2	1110 (65.3)	312 (65.1)	798 (65.4)		264 (70.2)	205 (64.9)	238 (72.1)	222 (65.9)	181 (53.1)	

^∗∗^Were you provided sufficient information about implant treatment procedures during your BDS program? (1) Yes. (2) No.

**Table 3:** Overall responses (*N*) to the 2nd question (A2) on perceived need of more information and its association with gender and academic levels.

Question		*N* (%)	Gender			Academic year					
			Male, *n* (%)	Female, *n* (%)	*P* value	1st, *n* (%)	2nd, *n* (%)	3rd, *n* (%)	4th, *n* (%)	5th, *n* (%)	*P* value
A2^∗∗^	1	1617 (95.1)	452 (94.4)	1165 (95.4)	0.216	361 (96.0)	295 (93.4)	301 (91.2)	330 (97.9)	330 (96.8)	0.001
	2	83 (4.9)	27 (5.6)	56 (4.6)		15 (4.0)	21 (6.6)	29 (8.8)	7 (2.1)	11 (3.2)	

^∗∗^Would you like more information about implant treatment procedures to be provided in the BDS curriculum? (1) Yes. (2) No.

**Table 4:** Overall responses (*N*) to the 3rd question (A3) on preferred source of more information and its association with gender and academic levels.

Question		*N* (%)	Gender			Academic year					
			Male, *n* (%)	Female, *n* (%)	*P* value	1st, *n* (%)	2nd, *n* (%)	3rd, *n* (%)	4th, *n* (%)	5th, *n* (%)	*P* value
A3^∗∗^	1	387 (22.8)	97 (20.3)	290 (23.8)	0.401	73 (19.4)	80 (25.3)	47 (14.2)	74 (22.0)	113 (33.1)	<0.001
	2	408 (24.0)	121 (25.3)	287 (23.5)		78 (20.7)	68 (21.5)	81 (24.5)	65 (19.3)	116 (34.0)	
	3	145 (8.5)	42 (8.8)	103 (8.4)		33 (8.8)	29 (9.2)	37 (11.2)	31 (9.2)	15 (4.4)	
	4	692 (40.7)	195 (40.7)	497 (40.7)		180 (47.9)	125 (39.6)	147 (44.5)	156 (46.3)	84 (24.6)	
	5	68 (4.0)	24 (5.0)	44 (3.6)		12 (3.2)	14 (4.4)	18 (5.5)	11 (3.3)	13 (3.8)	

^∗∗^From where would you like to get additional reliable information about dental implants? (1) Short-term CDE programs and workshops conducted by the implant companies. (2) One-year certificate or module-based courses conducted by colleges or trained implantologists. (3) Professional newsletters and books. (4) Dental consultants and specialists. (5) Study groups and the Internet.

**Table 5:** Overall responses (*N*) to the 4th question (A3) on preferred source of more information and its association with gender and academic levels.

Question		*N* (%)	Gender			Academic year					
			Male, *n* (%)	Female, *n* (%)	*P* value	1st, *n* (%)	2nd, *n* (%)	3rd, *n* (%)	4th, *n* (%)	5th, *n* (%)	*P* value
A4^∗∗^	1	332 (19.5)	90 (18.8)	242 (19.8)	0.201	64 (17.0)	91 (28.8)	67 (20.3)	55 (16.3)	55 (16.1)	<0.001
	2	513 (30.2)	149 (31.1)	364 (29.8)		90 (23.9)	85 (26.9)	95 (28.8)	107 (31.8)	136 (39.9)	
	3	667 (39.2)	176 (36.7)	491 (40.2)		163 (43.4)	103 (32.6)	139 (42.1)	135 (40.1)	127 (37.2)	
	4	188 (11.1)	64 (13.4)	124 (10.2)		59 (15.7)	37 (11.7)	29 (8.8)	40 (11.9)	23 (6.7)	

^∗∗^From where would you like to receive training on dental implants? (1) Short-term CDE programs and workshops conducted by implant companies. (2) One-year certificate or module-based courses conducted by colleges or trained implantologists. (3) Fellowship programs conducted by universities. (4) MSc programs (full time = 1 year, part time = 2 years).
